# A Dynamically Induced Phase Transition in Na_**4**_P_**2**_S_**6**_—Ultrafast Na^**+**^ Mobility Triggering Rotor Phase Formation

**DOI:** 10.1021/jacs.5c05339

**Published:** 2025-07-30

**Authors:** Katharina Hogrefe, Bernhard Gadermaier, Christian Schneider, Sebastian Bette, Bettina V. Lotsch, H. Martin R. Wilkening

**Affiliations:** † Institute of Chemistry and Technology of Materials, 27253 Graz University of Technology (NAWI Graz), Stremayrgasse 9, Graz 8010, Austria; ‡ 28326 Max Planck Institute for Solid State Research, Heisenbergstraße 1, Stuttgart 70569, Germany; § LMU München, Butenandtstraße 5-13, Munich 81377, Germany

## Abstract

The physical properties of any crystalline solid, such as the irregular movement of ions or atoms, are closely linked to its structure. Changes in local structure or local defect chemistry are typically attributed to changes in ion hopping. Conversely, one might also ask whether fast ionic diffusion can cause structural changes, finally initiating an overall phase transition. By using high-resolution ^23^Na and ^31^P nuclear magnetic resonance (NMR) carried out at temperatures as high as 650 °C, we show that changes of the local Na^+^ environment in the Na^+^-conducting model compound Na_4_P_2_S_6_ indeed precede the transition of the anionic framework from β-Na_4_P_2_S_6_ to the fast-conducting γ-phase. While rapid 2D Na^+^ diffusion governs ionic conductivity in the β-phase of Na_4_P_2_S_6_, the high-temperature γ-phase has been theoretically predicted and experimentally shown to be a rotor phase with high dynamics of both the mobile Na^+^ cations and the anionic framework. Here, we provide evidence that Na^+^ diffusion and the initial transformation of the Na substructure precede the transition of the P_2_S_6_ units to a rotating framework. NMR spectra and relaxation times of both ^23^Na and ^31^P reveal that rapid P_2_S_6_^4–^ motions occur in a molten Na^+^ substructure, but these motions do not influence Na^+^ hopping much. Hence, we suggest that Na^+^ hopping while first initiating the transformation to the rotor phase is indeed uncoupled from polyanion rotations at high temperatures. Our study provides a new perspective on the details governing phase transitions in fast-ion conductors and may lead to a deeper understanding of these phenomena.

## Graphical abstract

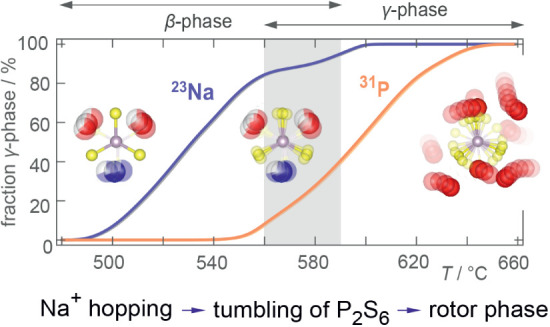

## Introduction

The surge in demand for ecologically friendly and energy-dense batteries calls for technological progress and a better fundamental understanding.^1^ Metal batteries, in which the highly mobile ion, such as Li^+^, is stored as a metal, are promising solutions but require chemically and electrochemically stable solid lithium electrolytes.^2^ The need to replace the critical resource lithium is pressing, especially in large and stationary energy storage systems. Na-ion batteries^3^ and, hence, Na^+^ ion conductors might serve as highly attractive alternatives.^4^ Especially if they consist of abundant elements, as they are more sustainable and cheaper compared to their Li counterparts.^5–9^ The development of Na-ion batteries equipped with solid electrolytes requires, however, fundamental work^10^ to understand the origins of fast Na^+^ ion hopping from the atomic length scale point of view.

As yet, apart from β-alumina,^11,12^ only a few solid Na^+^ conducting materials reached the application level in sodium batteries. Other intensively studied materials include, for example, NaSICON-type compounds,^9,13,14^ Na-bearing thiophosphates,^15–17^ and Na-halides,^18^ for example. Within these groups, the thiophosphates often suffer from air and moisture instabilities, which complicate their integration into electrochemical devices. The herein investigated Na_4_P_2_S_6_ shows, however, reversible hydration and dehydration due to the superior chemical stability of the P_2_S_6_-units.^19^

Sodium hexathiohypodiphosphate, Na_4_P_2_S_6_, is known to exist in three different polymorphs.^20–22^ The α-form is thermodynamically stable at room temperature and converts to the β-phase at 160 °C. This transition is accompanied by a redistribution of the Na ions from two sites in the α-phase to three sites in the β-phase. Interestingly, a β-like phase can be obtained at room temperature by a precipitation synthesis route, which yields a defect-rich, metastable structure that is arrested in the β-phase likely by stacking faults and crystallographic defects.^21^ In β-Na_4_P_2_S_6_, direct Na^+^ exchange between the Na1 site and the Na2/3 sites in the defect-rich layers is sterically hindered by the PS_3_-subunits and, thus, less likely to occur compared to rapid exchange between Na2 and Na3 (see Figure 1).

**Figure 1:**
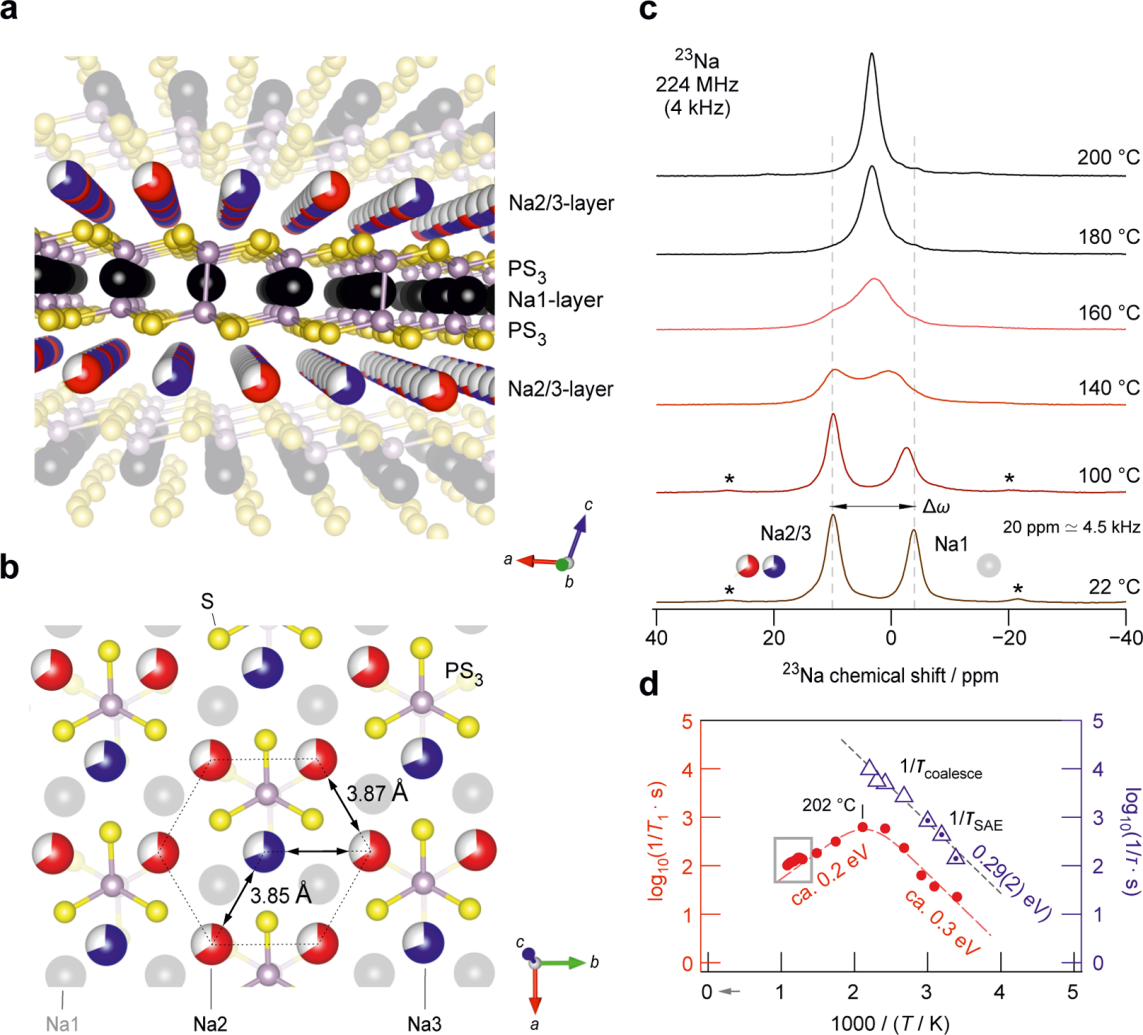
(a) Crystal structure of β-Na_4_P_2_S_6_ illustrating the layer-like arrangement of alternate Na2/3 and Na1/P_2_S_6_ layers. Na ions are shown in black (Na1), red, and blue spheres (Na2/3), P in purple, and S as yellow spheres. (b) Top view onto a Na2/3 layer shows the partially occupied sites that allow fast hopping processes within this layer; distances are indicated. An adjacent Na1/P_2_S_6_ layer is shown in the background. (c) ^23^Na MAS NMR lines recorded from 22 to 200 °C at a spinning speed of 4 kHz. Two separate lines evoked by the Na ions in the different layers, Na1/P_2_S_6_ and Na2/3, start to coalesce as the interlayer exchange rate rises with increasing temperature. Asterisks denote spinning side bands; 20 ppm corresponds to approximately 4.5 kHz. (d) ^23^Na relaxation rates, 1/*T*_1_, measured under MAS conditions at a Larmor frequency of 224 MHz. The relaxation rates derived from the total magnetization are shown by filled circles. At 202 °C, an asymmetric rate peak characterized by a low-*T* activation energy of ca. 0.3 eV, is indicated. Exchange jump rates 1/τ between the layers, as derived from both (i) NMR line coalescence and (ii) spin-alignment echo (SAE) NMR experiments (marked triangles), match perfectly and yield an activation energy of 0.29(2) eV. A magnification of the area marked by the gray box is shown in Figure 2c.

Recently, a third modification has been reported: the highly conductive γ-modification is stable above 580 °C and forms upon heating polycrystalline β-Na_4_P_2_S_6_.^22^ This high-temperature phase was identified by X-ray powder diffraction and pair distribution function analysis^22^ as a so-called rotor phase characterized by both rapid tumbling of the ethane-like P_2_S_6_-polyhedra and fast Na^+^ overbarrier hopping. For comparison, the β-γ phase transition causes an increase in electrical conductivity from 92(11) to 140(12) mS cm^–1^, which is accompanied by a drastic decrease in the activation energy from 0.56(5) eV to 0.09(2) eV. *Ab initio* molecular dynamics simulations identified the γ-phase as a plastic crystal with fast (jump) rotational motions of the P_2_S_6_^4–^ anions, indeed characterized by ionic conductivities in the order of 145 mS cm^–1^.^22^ Apart from impedance spectroscopy used to study macroscopic Na^+^ ion transport, little information about the exact translational Na^+^ hopping processes is available from experimental methods that use local nuclear probes. Moreover, the nature of the phase transitions has not been studied for a possible influence from Na^+^ ion dynamics.

This aspect is of special interest for phases with an active polyanion framework. For so-called rotor phases,^23^ there has been a long and ongoing debate on the possibility of correlated rotation of structural elements and the translation of the mobile cations.^23–28^ On the one hand, many simulations of different systems point to a certain degree of correlation or even perceived causation.^29^ As an example, Zhang et al. investigated Na_11_Sn_2_PnX_12_ (Pn = P, Sb; X = S, Se) and used joint time correlation analysis to find evidence for anion rotation coupled to long-range Na^+^ translational dynamics.^30^ Similar methods were applied to show enhanced Li^+^ diffusion as the paddle-wheel effect widens the bottleneck for cation jumps in β-Li_3_PS_4_.^26^ On the other hand, Sun et al. investigated BH_4_-substituted Li argyrodite and did not find any correlation between B–H bond motion and Li^+^ mobility by AIMD simulations.^31^ Jun et al.^32^ recently reported on simulations (AIMD and NEB) indicating that ion translation may favor anion reorientation in a way similar to the jump relaxation model of Funke.^33^ The latter was recently experimentally shown to describe that lattice distortions follow Li^+^ translation in (Li)­FePO_4_.^34^ Also, in the exceedingly high conducting Na_3–*x*_Sb_1–*x*_W_*x*_S_4_ no evidence was found confirming the paddle-wheel effect yet.^25^ Experimental proof on either side of the argument is, however, very limited. Additionally, it has not been shown clearly so far, whether the fast cation motion triggers rotations of the anionic framework, or whether the rotations of the framework allow for higher cation mobility. The similar time scale and quasi-simultaneous occurrence of both kinds of motion render this question a classic chicken-and-egg problem.

Temperature variable solid-state NMR provides unique insights into both local structural changes^35^ and variances in the dynamics^36,37^ of the NMR active nuclei. To study the dynamic and structural changes in Na_4_P_2_S_6_ from room temperature to 650 °C, we applied variable-temperature NMR line shape measurements and NMR spin–lattice relaxation rate measurements^38^ of both the ^23^Na nuclei (spin-quantum number *I* = 3/2) and the ^31^P spins (*I* = 1/2).^39^ By using a laser heating system in combination with a high external magnetic field, we were able to observe the changes in Na and P substructure upon the β-γ phase transition in Na_4_P_2_S_6_. In contrast to expectations based on X-ray diffraction, our study shows that the phase transition as seen by NMR is not abrupt. Instead, we find a continuous change first in the Na environment, followed by a transition of the P-bearing units. A strong decay of the ^31^P NMR spin–lattice relaxation times at temperatures higher than 600 °C underlines the rotor-phase character of γ-Na_4_P_2_S_6_. To the best of our knowledge, this is one of the first studies providing indications for a dynamically induced phase transition triggered by the mobile cations.

## Results

### Cation Translational Processes in β-Na_4_P_2_S_6_

The β-phase of Na_4_P_2_S_6_ is a layer-like structure characterized by alternate stacking of Na2/3 layers and Na1/P_2_S_6_ layers (see Figure 1a). The Na2 and Na3 sites are only partially occupied by two-thirds, which is ideal for rapid translational exchange within this layer. In the Na2/3 layer, each Na3 site is surrounded by six Na2 sites, while the Na2 site is surrounded by three Na2 and three Na3 sites (see Figure 1b).

In high-resolution ^23^Na magic angle spinning (MAS) NMR, β-like Na_4_P_2_S_6_ shows two distinct and well-separated NMR lines (Figure 1c). Although three distinct crystallographic sites are available (Figure 1a), only two lines are resolved because rapid Na^+^ exchange between the sites Na2 and Na3 already results in a coalesced line with an average chemical shift of 20 ppm at temperatures as low as 22 °C. Splitting of this line due to sluggish hopping between Na2 and Na3 is expected, if the two sites are magnetically inequivalent, at much lower temperatures and was absent even at a temperature as low as −83 °C (see Figure S1).

Effectively, each ^23^Na NMR line corresponds to a different layer, that is, the P_2_S_6_ layer (Na1) and the Na-rich layer (Na2/3 sites); see Figure 1a. Upon heating, increased Na^+^ exchange between these layers is expected. Indeed, at elevated temperatures the two lines gradually coalesce, finally resulting in a single NMR line with a width of only 200 Hz at 320 °C, see also Figure S2. This phenomenon gives direct access to the so-called *inter*layer exchange rate in β-Na_4_P_2_S_6_.^21^ The extracted rates^40^ 1/τ_coalesce_ are shown in Figures 1d and S2c. As expected from the kHz distance of the two signals on the frequency scale, they reveal a rather slow hopping process between the two groups of sites Na1 and Na2/3. As an example, at 180 °C, 1/τ_coalesce_ is in the order of 9.7 kHz, which translates into a diffusion coefficient *D*_NMR, inter_ in the range of 6 to 11 × 10^–12^ cm^2^ s^–1^, see Supporting Information. Raman measurements between 20 and 220 °C were inconspicuous while X-ray powder diffraction (XRPD) showed only a subtle change of the thermal expansion coefficient at approximately 140 °C (see Figure S3). Hence, the ^23^Na NMR line coalescence clearly shows that the feature observed by NMR is due to ion dynamics.

The interlayer hopping process results in temporal fluctuations of the local magnetic fields, i.e., chemical shifts, the Na^+^ ions are experiencing. Since ^23^Na (spin-quantum number *I* = 3/2) is a quadrupole nucleus, interactions with nonvanishing electric field gradients will also lead to electrical fluctuations if we consider jump processes between the electrically inequivalent sites Na1 and Na2/3. Hence, we used ^23^Na spin-alignment echo (SAE) NMR, which is particularly sensitive to slow exchange processes for which we expect a strong change in site-specific electric field gradients.^41^ While this method has predominantly been used for ^7^Li spins with moderate quadrupole moments *q*,^36,42–47^ it might be, to a limited extent, also applicable to the ^23^Na nucleus with a much larger *q* value.^48^

The resulting SAE NMR rates 1/τ_SAE_ are included in Figure 1d and S2c and yield an activation energy of 0.29(2) eV. Here, the rates 1/τ_coalesc_ derived from evaluating the coalescence phenomenon to which the NMR line shapes are subjected (see Figure 1d) excellently agree with those from SAE NMR. This slow, *quasi* 1D process characterizes interlayer hopping in the order of a few kHz. The total spin fluctuations probed by the ^23^Na NMR spin–lattice relaxation rates 1/*T*_1_ sense fluctuations on the MHz scale and hence cannot capture such a slow process. ^23^Na NMR spin–lattice relaxation in Na_4_P_2_S_6_ is expected to be dominated by fast intralayer dynamics involving only Na2/3 (see below). This interpretation excellently agrees with results from bond valence energy landscape calculations that point to 2D ion transport because of the strong interaction of Na1 with the P_2_S_6_ dumbbells.^21^

To investigate fast Na^+^ ion dynamics in β-Na_4_P_2_S_6_, we recorded ^23^Na NMR longitudinal, that is, spin–lattice, relaxation rates up to temperatures as high as 650 °C (Figure 1d). With this method we probe rapid spin fluctuations on the MHz time scale,^36^ i.e., we have access to the rapid Na^+^ intralayer exchange processes involving the Na2 and Na3 sites within the vacancy-rich layers. Any contribution from slow interlayer hopping is expected to be negligible here. As shown in Figure 1d, the spin–lattice relaxation rates 1/*T*_1_ pass through a diffusion-induced rate peak at 202 °C. Due to the limited number of data points, the exact shape of the peak is difficult to assess. Here, it seems that the peak is asymmetric, as indeed expected of a low-dimensional ion conductor.

On the low-*T* side, the rate peak yields an activation energy for intralayer jump diffusion of approximately 0.3 eV (Figure 1d). If we disregard correlation effects influencing this value,^36,49^ it describes the barrier for the elementary steps of ion hopping in the Na2/3 layer. The corresponding diffusion coefficient *D*_NMR, intra_ at the temperature where the peak appears is given by 5 × 10^–7^ cm^2^ s^–1^ (202 °C), see Supporting Information. For comparison, this Na^+^ hopping within the layers is by 5 orders of magnitude faster than Na^+^ exchange between them, see above. Though these findings result formally in an overall 3D diffusion behavior, the 5-orders of magnitude difference between intra- and interlayer jump diffusion renders the β-phase of Na_4_P_2_S_6_ an ionic conductor with anisotropic ion dynamics, at least if temperatures below 180 °C are considered. Ion dynamics is determined by rapid Na^+^ exchange within the vacancy-rich Na2/3 layers and a slow Na^+^ interlayer hopping rate.

The fast 2D intralayer translational process is also expected to dominate electrical conductivity (or impedance) measurements. Indeed, a specific ionic conductivity of 1.7 mS cm^–1^ obtained at 200 °C (Figure S3) translates into a diffusion coefficient *D*_σ_ of approximately 3 × 10^–8^ cm^2^ s^–1^ (see SI), which is only slightly lower than *D*_NMR, intra_ as deduced from NMR. Although slow, the onset of interlayer hopping might influence intralayer transport of the Na^+^ ions. Indeed, at around 140 °C, i.e., at the beginning of significant coalescence, we recognize a kink in the thermal expansion coefficient and a decrease of the activation energy of the Arrhenius behavior of the bulk conductivity describing overall ion dynamics in Na_4_P_2_S_6_, see Figure S3. In general, solid-state diffusion coefficients, extracted from impedance data in a certain frequency range are additionally affected by possible interfacial resistances from grain boundary regions. Hence, apart from other reasons, such as interfacial effects and even influences from correlation effects, we expect deviations between *D*_NMR, intra_ and *D*_σ_.^38^ The similarity of *D*_NMR, intra_ and *D*_σ_ indicates that the same process is being observed by NMR and impedance. For further discussion, we refer to the Supporting Information.

### Transition from β-Na_4_P_2_S_6_ To γ-Na_4_P_2_S_6_

The phase transition from β-Na_4_P_2_S_6_ to γ-Na_4_P_2_S_6_ can be studied by both ^23^Na MAS NMR and ^31^P MAS NMR line shape measurements (Figure 2a,b). At first, we will discuss the changes seen in ^23^Na MAS NMR. Between 180 and 490 °C a single line is observed (see Figure 3) and up to 590 °C, a second line emerges and gains intensity on the expanse of the former line. As no phase transition could be detected by XRPD analysis (see Figure S6) and no further change of the ^23^Na line is observed up to 650 °C, we believe that the Na sublattice experiences the β-γ phase transition at a much lower temperature due to dynamic effects. Hence, between 490 and 590 °C, the spectra reveal a gradual transition from the β-form to the γ-phase marked by the emergence of a new ^23^Na NMR line centered at −1.45 ppm (see Figure 2b). As shown in Figure 2b, these two lines representing Na^+^ in the two phases of Na_4_P_2_S_6_ coexist over a relatively large temperature range, which we can only partly ascribe to the temperature gradient in the sample (see Experimental Section). Obviously, the phase transition is controlled by kinetic hindrance rather than solely dictated by thermodynamics. The integrals of the two phase-specific lines show that the transition progresses continuously over a temperature range covering almost 100 °C (see the temperatures indicated in Figure 2b) – temperature differences within the rotor account for approximately 43 °C.

**Figure 2:**
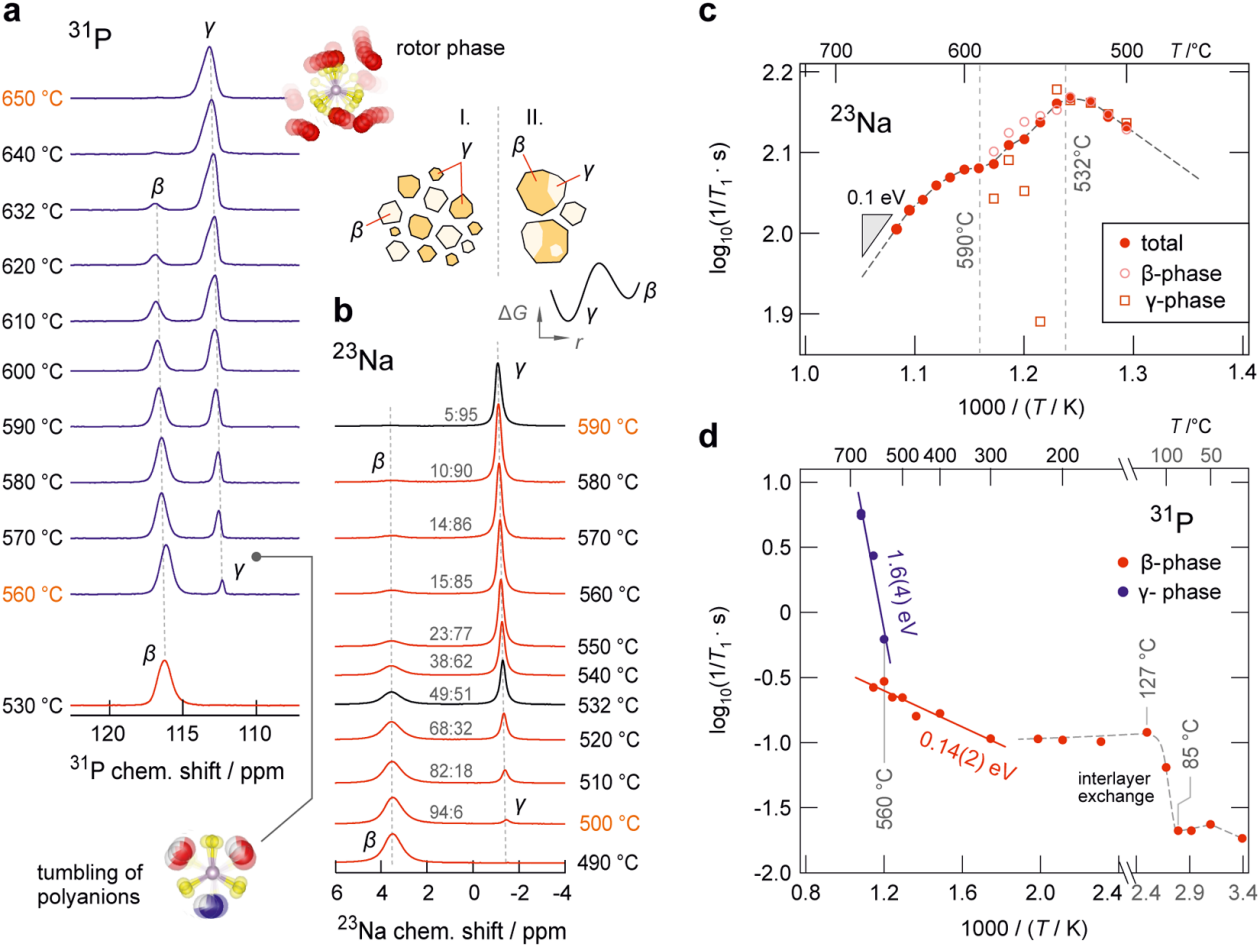
β-γ phase transition in Na_4_P_2_S_6_ as seen by NMR: (a) ^31^P NMR lines and (b) ^23^Na NMR spectra recorded at temperatures where the phase transition takes place. Below 530 °C, the ^31^P MAS NMR spectra show a single, slightly anisotropic line. With increasing temperature, a second line emerges with an ever-increasing area fraction until the previous line is no longer visible at 650 °C. The ^23^Na MAS NMR spectra recorded from 490 to 590 °C show the coexistence of the two phases of Na_4_P_2_S_6_ over a large temperature range, see illustration on top of (b) referring to a given temperature *T*. Ratios indicate the relative area fractions of the two lines. (c) Temperature dependence of the ^23^Na NMR spin–lattice relaxation rates above 490 °C. Filled circles show the overall relaxation rates and empty symbols represent the separate line-specific relaxation rates of the β- and the γ-signal. The ratio of the area fractions of the two signals contributing to the spectra at 532 °C is 1:1. Above 590 °C, ^23^Na NMR relaxation in the γ-phase controls the total rate. (d) ^31^P NMR spin–lattice relaxation rates recorded at temperatures of up to 650 °C. The sudden increase of the ^31^P NMR relaxation rate between 85 and 127 °C is associated with the onset of interlayer Na^+^ exchange (see Figure 1c). While the ^31^P NMR spin–lattice relaxation rates in the β-phase show a weak temperature dependence (0.14 eV) above 300 °C, the motion-induced rates in the γ-phase, observed above 560 °C, increase abruptly with temperature. The apparent activation energy of 1.6 eV is affected by the phase transition and an increasing fraction of the γ-phase.

**Figure 3:**
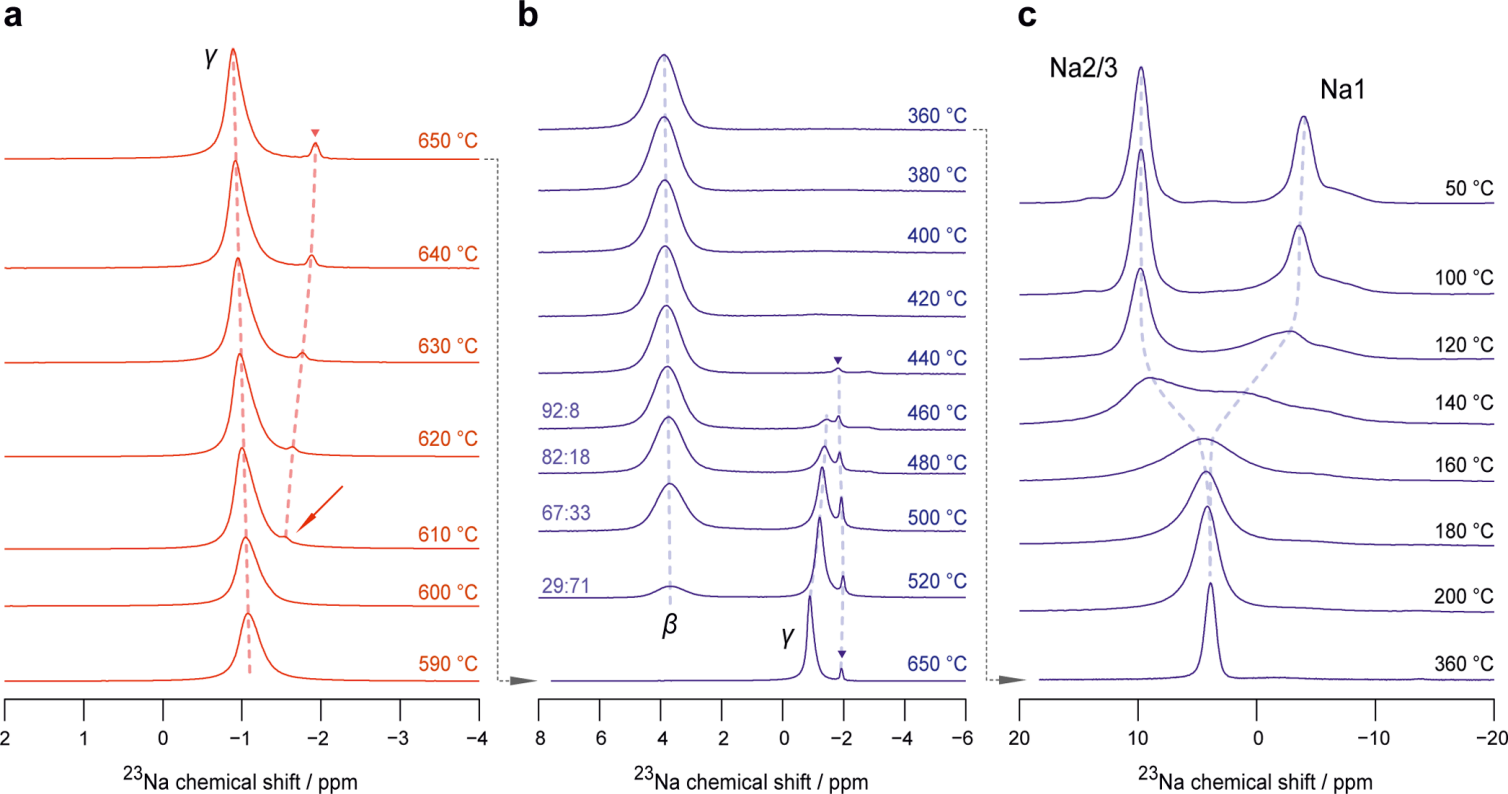
(a) ^23^Na MAS NMR spectra recorded during heating from 590 to 650 °C and (b, c) during cooling back to 50 °C. At 610 °C, a minor line, corresponding to a forming side-phase appears at −1.5 ppm, see arrow. During cooling, the γ-phase converts back to the β-modification and both phases coexist between 520 and 460 °C; ratios denote relative phase fractions. At temperatures below 420 °C, the previously formed side-phase is no longer visible indicating that Na_4_P_2_S_6_ is fully reformed. Cooling the β-phase further down leads to a significant decrease in interlayer Na^+^ exchange. The coalesced line splits up into two separate resonances (see Figure 1c).

This finding is in contrast to that observed by *in situ* X-ray diffraction and DSC revealing an abrupt phase transition at 580 °C.^22^ The exact transition temperature may depend on the exact defect chemistry that differs from batch to batch. Temperature-dependent XRPD measurements reveal a phase transition temperature of approximately 600 °C for this particular sample (see Figure S6). While X-ray diffraction and DSC detect a temporally averaged picture of the crystal lattice at each temperature, ^23^Na NMR is a local probe and senses this phase transition already at temperatures as low as 500 °C (see Figure 2). At this temperature, parts of the ^23^Na nuclei already experience a local magnetic environment identical to that of the γ-phase for which the activation energy for translational ion dynamics is as low as 0.1 eV, see Figure 2c. Such findings add detailed information not only to the onset of faster Na^+^ ion dynamics but also to the beginning of the high-temperature phase transition of Na_4_P_2_S_6_.

To support our idea of two coexisting phases in Na_4_P_2_S_6_, we analyzed the ^23^Na MAS NMR spectra in more detail. The new ^23^Na NMR signal appears at a higher field and forms at the expense of the coalesced Na NMR signal of the β-phase (Figure 2b). The clear separation of these two lines, without any intensity in between, indicates that these two lines stem from two distinct Na^+^ environments without significant exchange; otherwise, the lines would be affected by coalescence effects as shown for the *inter*layer exchange (*vide supra*). For Na^+^ in the on-average cubic γ-phase, a separate diffusion mechanism seems to occur. Thus, the formation of regions of γ-Na_4_P_2_S_6_ is marked by the appearance of a different Na^+^ local environment. The coexistence of both phases, as seen by ^23^Na MAS NMR could give rise to two subpopulations that undergo independent spin–lattice relaxation without a significant exchange between the respective environments. The latter assumes no effective spin-diffusion between these two reservoirs. Indeed, two ^23^Na NMR spin–lattice relaxation rates determine the overall longitudinal recovery of the magnetization above 500 °C (Figure 2c, see below).

Multiple origins could explain the two coexisting magnetically inequivalent ^23^Na sites, as has also been discussed for the phase transition kinetics of LiNaSO_4_ by Shakhovoy et al.,^50^ but cannot be ascribed solely to temperature gradients within the sample. The NMR response might point to a metastable mixture of spatially separated crystallites whose one-by-one transformation depends, besides other factors, on their individual defect structure,^51^ see illustration I. in Figure 2. Alternatively, domains of the β-form and the γ-modification could be present in the same grain (see illustration II. in Figure 2) as would be in line with an interface-controlled growth process.^51^ In such a case, only the fraction of ions near the β-γ grain boundary regions would participate in Na^+^ exchange processes resulting again in marginal coalescence effects. Worth noting, the two ^23^Na MAS NMR signals do not even change as a function of time if the sample is kept at the same temperature for a longer duration (see Figure S5). This finding again suggests sluggish phase transition kinetics. Likely, kinetic effects,^50^ dominate this transition since the β → γ conversion shows a rather high difference in Gibbs free energies. Furthermore, the temperature gradient in the sample and the rather high forces acting on the sample spun at 4 kHz during the measurement might indeed affect this phase transition with a rather high volume change. Finally, the changes in local environments sensed by the ^23^Na nuclei could also be associated with the onset of the increasing rotational amplitudes of the P_2_S_6_^4–^ anions.^22,32^ This onset might proceed over a relatively large temperature range.

As mentioned above and in agreement with the two ^23^Na NMR signals observed between 500 and 590 °C, we found biexponential spin–lattice relaxation transients in this temperature range. Figure 2c shows a magnification of the 1/*T*_1_ rates at temperatures higher than approximately 500 °C—the temperature at which the Na NMR signal of the γ-phase is first observed. As assumed for a β-γ phase mixture, we were able to determine and analyze phase-specific 1/*T*_1_ separately. Although the rates show strong fluctuations above 532 °C (see empty symbols), they differ by less than a factor of 1.8. Above 590 °C, a single spin–lattice relaxation rate governs spin–lattice relaxation, which is solely controlled by the ^23^Na spins in the γ-phase (Figure 2b). Even though the ^23^Na NMR rate in this narrow temperature window appears to have two maxima, they do not necessarily represent diffusion-induced rate peaks. Here, below 590 °C we interpret changes in ^23^Na NMR spin–lattice relaxation as being caused by a smooth transition from 1/*T*_1_ rates dominated by the β-phase to rates dominated by the γ-phase. This idea is in line with the evolution of the ^23^Na MAS NMR spectra (Figure 2b): at 532 °C, the temperature at which the rate peak appears, the ^23^Na NMR lines indicate a β-γ ratio of 1:1 (Figure 2a). Accordingly, the NMR rate peak at 532 °C can be interpreted as structure-change induced. Similarly, the shoulder seen at approximately 600 °C reflects structural and dynamic changes to which the ^31^P spins are subjected, as will be discussed below. Here, these temporal fluctuations are indirectly sensed by the ^23^Na spins. Above 620 °C the ^23^Na NMR 1/*T*_1_ rates point to an activation energy of 0.1 eV, which is in excellent agreement with that describing fast ion transport in the γ-Na_4_P_2_S_6_ rotor phase as found earlier by using impedance spectroscopy.

Upon cooling, all changes in the ^23^Na NMR spectra are fully reversible, see Figure 3. The fact that the ^23^Na MAS NMR lines are slightly narrower at ambient can be traced back to *in situ* annealing of the sample (see Figure 3). After exposure to high temperatures during the NMR measurements defects were healed (see also Figure S6) leading to a smaller distribution of chemical environments and hence narrower lines. The transition of the ^23^Na NMR signal above 500 °C shows a hysteresis in the cooling cycle. Likely, this hysteresis results again from sluggish kinetics^50^ of the β-γ phase transition. A similar behavior has been observed for Na_3_PO_4_, where the γ-phase forms at the expense of the α-phase over a range of more than 100 °C as detected by XRPD.^52^ Here, similar to Na_3_PO_4_, the large volume difference of the respective unit cells (4.9%)^22^ increases the energy barrier for nucleation of the β-phase in the γ-phase upon cooling.

Apart from the ^23^Na spins, also the ^31^P nuclei (*I* = 1/2) of the polyanions can serve as spies to collect information on structural changes and dynamic features, as shown earlier by some of us for Li_6_PS_5_X (X = Cl, Br, I).^53^ In general, tumbling of P–P dumbbells, locked in the polyhedra of the anion framework, or coupling to highly mobile ions like Li^+^ or Na^+^ may cause changes in the ^31^P NMR spin–lattice relaxation behavior.^39,53,54^ Consequently, we also analyzed ^31^P MAS NMR lines and ^31^P NMR spin–lattice relaxation rates as a function of temperature. Here, up to 530 °C, only a single, slightly anisotropic ^31^P NMR line is observed (Figure S4). At even higher temperatures, a second line emerges and gains in intensity with increasing temperature (Figure 2a). This line is attributed to that belonging to ^31^P in the γ-form of Na_4_P_2_S_6_. Similar to ^23^Na NMR, the ^31^P spies probe a gradual, rather than an abrupt, phase transition, which is only in part attributed to the expected temperature gradient in the sample. However, in contrast to ^23^Na NMR, the transition is shifted toward higher temperatures (560 to 650 °C compared to 500 to 590 °C). Altogether, ^23^Na and ^31^P NMR cover a temperature range from 500 to 650 °C where the full β-to-γ transition takes place. X-ray diffraction and DSC detects the β → γ transition half way,^22^ that is, at approximately 600 °C, see above. Though its sensitivity to changes at the atomic scale, we believe that results from ^31^P NMR, probing the arrangement of the P_2_S_6_^4–^ framework units, are more comparable to results from XRPD detecting a macroscopic average of the Na_4_P_2_S_6_ crystal lattice.

Notably, the shape of the ^31^P MAS NMR line of γ-Na_4_P_2_S_6_ is not only anisotropic (610 °C) but also broadens with increasing temperature, see, e.g., the spectra recorded above 600 °C. This feature might be ascribed to the various rotational motions of the P_2_S_6_^4–^ units in the rotor phase that is established at sufficiently high *T*. Starting at 610 °C, a small amount of another, minor phase is formed which is indicated by the appearance of a tiny ^23^Na NMR line that is also seen in ^31^P MAS NMR (Figure S4). The newly formed side phase includes both Na and P species. Its formation is fully reversible as no more traces of this phase are seen by NMR when cooling the sample back to room temperature (see Figure 3).

To study the role of phosphorus and the fingerprint of a rotor phase above 560 °C, we complement our study by measuring ^31^P NMR 1/*T*_1_ rates over a wide temperature range (Figure 2d). At temperatures around ambient, the ^31^P NMR spin–lattice relaxation is very slow with relaxation rates 1/*T*_1_ in the order of 0.02 s^–1^. Such low rates can be rationalized by the fact that ^31^P is sterically fixed in the bulky dumbbell-shaped P_2_S_6_^4–^ polyanions. However, between 85 and 127 °C, the ^31^P NMR 1/*T*_1_ rates suddenly increase 4-fold. Exactly in this temperature range, Na^+^ ion exchange between the layers significantly increases and causes the two distinct Na NMR lines to coalesce. Hence, it is tempting to speculate that ^31^P NMR spin–lattice relaxation indirectly senses this interlayer Na^+^ exchange. As the ethane-like P_2_S_6_^4–^ units separate the Na2/3 layers and the PS_3_^2–^ units are located just in between the Na2/3 and Na1 layers (Figure 1a), the additional magnetic fluctuations caused by Na^+^ interlayer hopping could indeed affect ^31^P NMR 1/*T*_1_ relaxation, as found here. We exclude that the P-units show a significant tumbling motion or C_3_-rotation influencing the Na^+^ dynamics in the investigated temperature range, because the line spectra of ^31^P do not change. This means that in the temporal average, the magnetic environment of phosphorus does not change, which would be expected for beginning or intensifying rotations of the anionic framework. Another argument against the existence of a paddle-wheel effect concerns the absent exchange of Na^+^ between the different spin-reservoirs upon the β-γ phase transition (see above). If the C_3_-rotation of the P_2_S_6_^4–^ dumbbells influenced the phase transition, we would expect to see ionic hopping between these two Na-environments, as the dumbbells’ motion shoves the Na^+^ between different phases. This, however, can be excluded by our experimental data.

Between 130 and 300 °C, the ^31^P NMR 1/*T*_1_ rate is almost independent of temperature. Above 300 °C, it slightly increases with *T*, following Arrhenius behavior with 0.14(2) eV. For comparison, at 500 °C the new Na sites in the γ-like regions become evident and at temperatures higher than 530 °C, the new ^31^P NMR signal of γ-Na_4_P_2_S_6_ appears. Finally, at *T* > 560 °C, the corresponding ^31^P NMR magnetization transients give rise to two site-specific 1/*T*_1_ rates, 1/*T*_1,fast_ and 1/*T*_1,slow_. 1/*T*_1,fast_ belongs to the ^31^P NMR signal of γ-Na_4_P_2_S_6_ and shows a strong change with temperature (1.6 eV). As an example, already at 600 °C, this rate is 10 times higher than 1/*T*_1,slow_ associated with ^31^P in β-Na_4_P_2_S_6_. This steep rise suggests that a different relaxation mechanism is now at play in γ-Na_4_P_2_S_6_. Based on recent findings reported in the literature^22^ we suggest that it is controlled by the rapid rotational jumps of the P_2_S_6_-units, as expected for this rotor phase mentioned above.

## Discussion

Directly observing different nuclei in a time-dependent and site-specific manner is one of the most powerful features of nuclear methods such as high-resolution NMR and provides unique insights into complex structural and dynamic processes in crystalline and amorphous solids. Using ^23^Na MAS NMR, we show that ionic transport in layer-structured Na_4_P_2_S_6_ occurs in three dimensions. While simulations pointed to 2D-diffusion, only including the Na2 and Na3 sites of the Na-rich layer, the coalescence phenomenon of the NMR spectra unequivocally reveals that the Na1 site participates in diffusional transport. Results from spin-alignment echo NMR, sensitive to exchange between chemically different sites, match perfectly with the exchange rate derived from the coalescence and verify 3D ion transport in the β-phase of Na_4_P_2_S_6_. While this interlayer exchange is clearly visible, it shows rather slow dynamics compared to the fast 2D-like diffusion along the Na2/3 layer. For this intralayer Na^+^ ion hopping process, the jump rates are orders of magnitude higher (10^9^ s^–1^) as seen by a rate maximum in ^23^Na spin–lattice relaxation at 202 °C. The corresponding NMR diffusion coefficient perfectly agrees with that deduced from impedance spectroscopy, revealing that intralayer Na^+^ hopping, i.e., 2D ion conduction, governs long-range ionic transport in this phase. Our data thus present a very detailed picture of ionic motion in β-Na_4_P_2_S_6_.

Monitoring the evolution of ^23^Na and ^31^P NMR spectra over the temperature range for the *β-γ*-phase transition reveals unprecedented details about the nature of this transition. The ^23^Na NMR lines uncover a change in the local Na environment well below the reported phase transition temperature. This finding shows that the Na^+^ ion substructure transforms before the rigid framework, as summarized in Figure 4. This transition occurs over a wide temperature range of 100 °C. We postulate that this change in the local environment of Na^+^ is dynamically induced, meaning that the sodium ions occupy a new magnetically averaged site by accessing a new diffusion pathway. This pathway is unique to the γ-phase, as no exchange between the β- and the γ-specific line is observed in the NMR spectra; see Figure 2b. The idea that translational motions induce or initiate the transformation of a material into a new form has recently been presented for Li_2_OHCl,^55^ which undergoes an orthorhombic-to-cubic phase transformation slightly above 30 °C.

**Figure 4:**
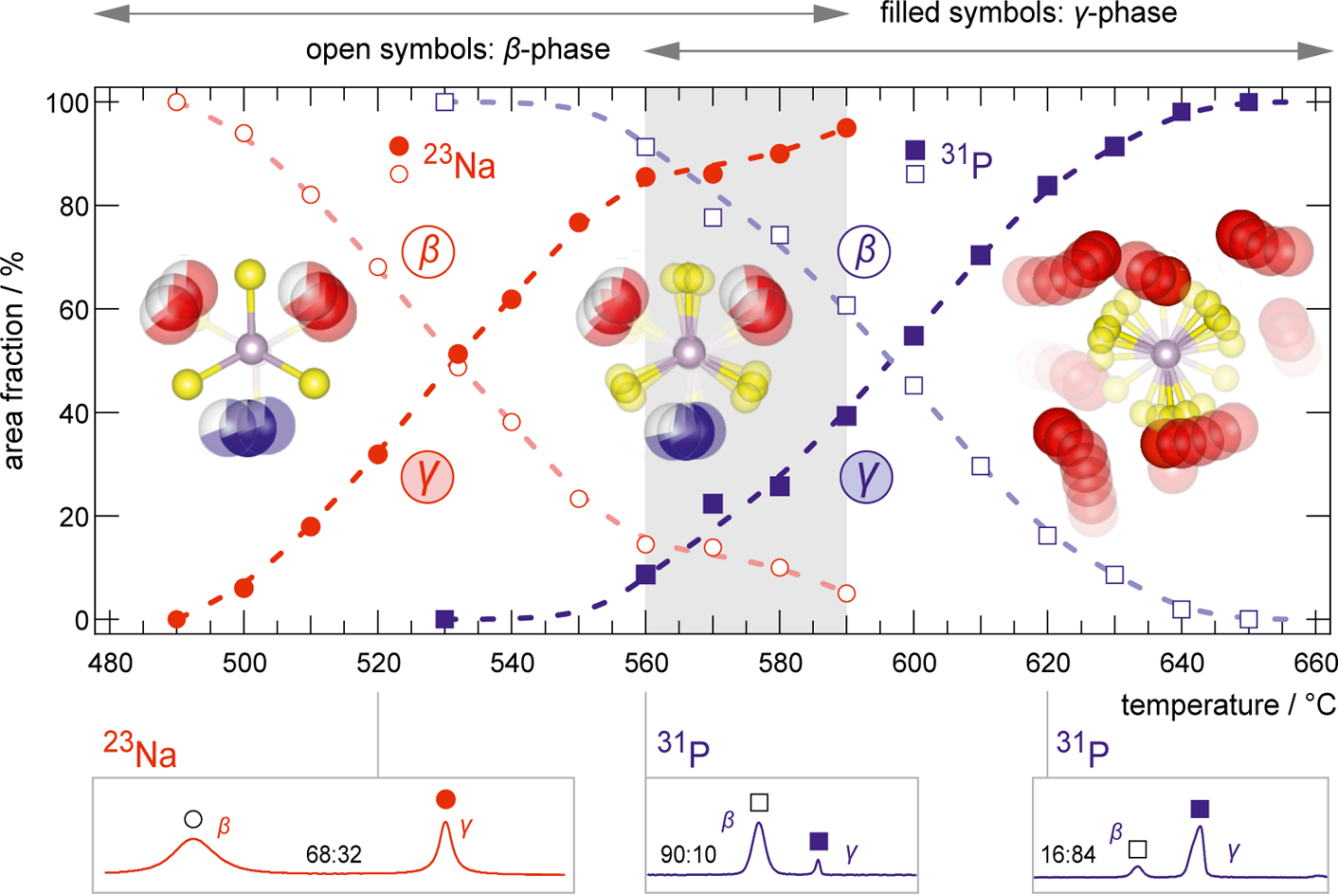
Relative area fractions of the ^23^Na and ^31^P MAS NMR lines over the whole β-γ phase-transition temperature range. MAS NMR spectra recorded at the indicated temperatures are shown at the bottom. Starting at 500 °C, the ^23^Na spectra reveal the onset of the phase transition by the emergence of a new line that gradually increases until dominating the spectra. Before this process is completed, the ^31^P spectra reveal a similar picture: a new line emerges and grows with temperature. Importantly, these two processes are likely linked and the fast Na-diffusion kick starts the rotation of the rotor phase as sensed by an ever-increasing fraction of ^31^P nuclei. In the structural model, Na-ions are shown as red and blue spheres, while the P_2_S_6_-units are depicted with purple (P) and yellow (S) spheres. Initially, only Na-ions move within the structure. At 560 °C, the P_2_S_6_-units begin to vibrate until the structure becomes a rotor phase.

While the ^23^Na spectra change drastically up to 590 °C, the anionic framework observed via ^31^P spectra is not affected up to 532 °C. Here, as the γ-phase starts to dominate ^23^Na NMR spin–lattice relaxation, a new ^31^P NMR line emerges. Accordingly, the structural framework including the P–S-units is only affected when the faster Na-ions of the local γ-structure govern overall diffusion of the mobile ions. Only after the new structural framework is formed, ^31^P NMR longitudinal relaxation accelerates abruptly, as the phase finally converts to a rotor phase. Our observations in this system shed light on the elementary steps of the phase transition to a rotor phase. We reveal the melting of the cation substructure first preceding and then proceeding with the steric liberation of the anion framework. These subsequent processes show that the fast rotational jumps in the rotor phase take place in the presence of a molten Na^+^ substructure but are uncoupled from fast Na^+^ translational motions in the temperature range of the phase transition. Up to 650 °C, NMR does not see evidence of a paddle-wheel or cogwheel mechanism.^23^ We cannot determine these mechanisms for a fully converted γ-structure in Na_4_P_2_S_6_ by solid-state NMR as we reach hardware limitations for observing such fast processes. However, the abrupt change in the P relaxation and the strong *T*-dependency point to fast dynamic processes of the anionic framework. We interpret the succession of events as the rapid Na^+^ translational motions kick-starting the conversion to a rotor phase. In the investigated *T*-frame, we do not observe a bidirectional correlation, i.e., the rotations of the polyanions do not significantly influence Na^+^ displacements.

The situation might be different for lower temperatures, where we cannot exclude that Na^+^ motions are correlated to tumbling or rotating of anion framework. In this temperature range, slight reorientations such as small-angle rotational jumps, might indeed influence the forward–backward jump processes to which the ions are subjected in an asymmetric double-well potential, as described by the classical jump-relaxation model of Funke^33^ and by recent suggestions of Ceder and coworkers,^32^ for example. Appropriate reorientations of the polyanion directly succeeding a Na^+^ jump, that is, a relaxation of the framework, would decrease the probability of a backward jump and increase the number of successful ionic displacements. The same cation–anion interplay has been suggested for Li_2_OHCl,^55^ see above. In general, such fundamental observations of the different dynamic steps leading to fast long-range transport are likely also present in other types of ion conductors. They highlight the important interplay of the mobile and the (polyanion) framework to understand overall cation dynamics. Besides pure thermodynamic factors, the nature of the phase transition seems to be influenced even by these dynamic variables.

## Conclusion

We investigated structural and dynamic features of the Na-ion conductor Na_4_P_2_S_6_ in its crystallographic β-phase and across the β-γ phase transition using ^31^P and ^23^Na MAS NMR. ^23^Na MAS NMR reveals highly anisotropic but nevertheless, three-dimensional ionic transport in layer-structured β-Na_4_P_2_S_6_. Combined analysis of ^31^P and ^23^Na shows that the highly mobile Na ions gain access to a new diffusion pathway at temperatures preceding the β-γ-phase transition, as seen by XRPD and ^31^P MAS NMR analysis. ^31^P NMR longitudinal relaxation accelerates abruptly, as the phase finally converts to the γ-phase, a rotor phase. The sequence of events during heating, i.e. melting of the Na substructure, followed by the steric liberation of the anion framework, shows an unprecedented insight into the microscopic processes of the formation of a rotor phase and suggests that fast rotational jumps in the rotor phase are uncoupled from fast Na^+^ translational motions.

## Experimental Section

### Sample Preparation and Characterization

Na_4_P_2_S_6_ was prepared from its hexahydrate form Na_4_P_2_S_6_·6H_2_O by heating the hydrate for 24 h at 100 °C under a dynamic vacuum (Büchi furnace). Na_4_P_2_S_6_·6H_2_O was prepared via a precipitation route starting from Na_2_S·9H_2_O (75 g, Aldrich, > 99.99%), which was dissolved in deionized water (100 mL). While stirring the solution, PCl_3_ (6.6 mL, Arcos, 99%) was added dropwise to control the strong exothermic reaction of PCl_3_ with water. The cloudy solution was stirred for 20 min and cooled down using an ice water bath until white, flakey crystals of Na_4_P_2_S_6_·6H_2_O precipitated. The suspension was allowed to warm up to room temperature while stirring and then stored for 1 to 2 h before cooling it down to 4 °C in a refrigerator to completely precipitate the hexahydrate overnight. The powder was then separated from the mother liquid and recrystallized from a water:ethanol solution (75:25) at 80 °C. During recrystallization, large, white, hexagonal crystals formed.

### X-Ray Powder Diffraction and Raman Spectroscopy

X-ray powder diffraction (XRPD) patterns for temperature-dependent *in situ* data were collected at room temperature on laboratory powder diffractometers in Debye–Scherrer geometry (Stadi P-Diffraktometer (Stoe), Cu–K_α1_ or Mo–K_α1_, respectively, radiation from primary Ge(111)-Johann-type monochromator, triple array of Mythen 1 K detectors (Dectris). The sample was sealed in a 0.5 mm quartz glass capillary (Hilgenberg) which was spun during the measurements. A hot and cool air blower (Cobra 700, Oxford Cryosystems) was used to heat it to 220 °C. The heating was performed in 10 °C steps until 100 °C and in 5 K steps until 220 °C using a heating rate of 5 K/min and an equilibration time of 5 min. XRPD patterns were recorded subsequently in a 2θ range from 0.0° to 110.0° applying a total scan time of 30 min. During the temperature-dependent *in situ* XRPD measurements using the device equipped with Mo–K_α1_ radiation, Raman spectra were collected using a Raman spectrometer (HORIBA, iHR 320) with a length of 320 mm equipped with an air-cooled CCD detector, a 633 nm HeNe laser (17 mW), a 900 gr/mm grating and a fiber optic Raman probe for noncontact remote measurements. The capillary, the hot air blower, and the Raman probe were installed into the X-ray powder diffractometer in such a way, that both the X-ray, the Raman laser, and the hot air stream hit the capillary in the same position, as described elsewhere.^56^ The program TOPAS 6.0 ^57^ was used for the analyses of the XRPD patterns. For the data analyses, LeBail fits^58^ applying the fundamental parameter approach of TOPAS,^59,60^ the lattice parameters of Na_4_P_2_S_6_,^21^ and Chebyshev polynomials of sixth order for modeling the background were employed.

### MAS NMR

^23^Na MAS NMR line shapes were recorded using a Bruker spectrometer connected to an 850 MHz magnet (Bruker), corresponding to a nominal magnetic field of 19.97 T. The Larmor frequencies of ^23^Na and ^31^P were 224.9 and 344.1 MHz, respectively. The sample was spun at 4 kHz while a variable-power pulsed CO_2_ laser (*t*_ON_ = 250 μs, *t*_OFF_ = 750 μs), guided by an optic fiber and a mirror, was used to adjust the temperature between 50 and 650 °C by direct illumination of the rotor insert. The sample temperature was determined by referencing the laser power to the NMR chemical shift of ^79^Br of a KBr sample measured under the same conditions.^61^ As only one side of the rotor is heated by the Laser, temperature gradients of up to approximately 46 °C (0.05 °C/K)^62,63^ are expected up to 650 °C. The sample was placed in an all-ceramic insert, which is slightly larger than the laser footprint, which was positioned in a ZrO_2_ ceramic rotor. The laser power level, corresponding to the desired temperature, was adjusted and kept constant during the measurement at each temperature. The extremely fast energy transfer via laser results in extreme heating rates, and the sample typically reaches the set temperature after a few seconds.^64^ After setting a different laser power, we performed consecutive line measurements to detect thermal stability and recorded the lines and rates using additional scans. For the ^23^Na MAS NMR measurements, a pulse length of 3.8 to 7.5 μs (90° pulse) was used to excite the spin system. Typically, 32 scans, separated by a recycle delay of 2 s, were accumulated to obtain free induction decays with satisfactorily high signal-to-noise ratios. The ^23^Na MAS NMR spectra were referenced to the ^23^Na NMR signal of crystalline NaCl (Aldrich, 0 ppm). CaHPO_4_ (Aldrich, 99%), referenced to the NMR chemical shift of ^31^P in H_3_PO_4_, served as a secondary (solid) reference to determine the isotropic NMR chemical shifts of the corresponding ^31^P MAS NMR spectra. To evaluate the phase fractions, i.e., the area under the NMR signals, we used Gaussian and Lorentzian functions to approximate the shape of the lines and used the QUADcentral model implemented in the topspin software package “sola”.

### Time-Domain NMR

Variable-temperature ^23^Na and ^31^P NMR spin–lattice relaxation rates 1/*T*_1_ in the laboratory frame under MAS conditions were recorded at a magnetic field of 19.97 T, see above. We applied the saturation recovery pulse sequence^65^ with variable delays (vd) to record longitudinal magnetization transients. Pulse lengths ranged from 3.8 to 7.5 μs (300 W) and from 1.3 to 4.7 μs (250 W) for ^23^Na and ^31^P, respectively. In the case of ^31^P NMR, vd-lists with a delay of up to 500 s were selected, while for ^23^Na a list with values ranging from 100 μs to 1 s was sufficient to record the full recovery of the magnetization. A recycle delay time of 5 × *T*_1_ was used to ensure full relaxation between each scan. We extracted the characteristic spin–lattice relaxation rates by fitting the transients with stretched exponential functions. ^23^Na SAE NMR decay curves were recorded with a Bruker Avance III (7 T) spectrometer (Bruker). To generate the stimulated echoes, we employed the three-pulse sequence introduced by Jeener and Broekaert.^41^ Echoes were recorded at fixed preparation time (15 μs) and variable mixing time.^42^ The π pulse length was set to 3.5 μs. A phase cycling with 32 entries was applied to suppress unwanted coherences.^45^

### Impedance Spectroscopy

For impedance measurements, the sample was pelletized by uniaxial cold-pressing in an argon-filled glovebox (GS, H_2_O < 1 ppm, O_2_ < 1 ppm). In the same box, the parallel surfaces of the pellet were sputter-coated by a 50 nm thick layer of gold (Leica Magnetron sputter coater). Impedance measurements were performed using a Novocontrol concept 80 setup. The pellet is continuously subjected to a stream of freshly evaporated nitrogen of defined temperature whereby the sample temperature is controlled and set between −100 and 300 °C. At each temperature, the impedance is measured at logarithmically even spaced frequencies between 10 mHz and 10 MHz using a voltage amplitude (root-mean-square value) of 100 mV.

## Supplementary Material



## Notes

Additional experimental procedures, analysis, low-temperature ^23^Na MAS NMR, Raman spectroscopy, impedance spectroscopy, additional ^23^Na and ^31^P MAS spectra, and high-temperature XRPD. Also contains Figures S1–S6 (PDF)
