# Supplementary material for: A Dynamically Induced Phase Transition in Na 4 P 2 S 6 —Ultrafast Na + Mobility Triggering Rotor Phase Formation
Source: J Am Chem Soc. 2025 Jul 30;147(32):28799–809. doi: 10.1021/jacs.5c05339 (PMC12356594; doi:10.1021/jacs.5c05339)
Supplement: Supplementary file 1 [file ja5c05339_si_001.pdf]

Supporting Information

A Dynamically Induced Phase Transition in $Na_4P_2S_6$ – Ultrafast Na^+ Mobility Triggering Rotor Phase Formation

Katharina Hogrefe,[#,1] Bernhard Gadermaier,[#,1*] Christian Schneider,[2] Sebastian Bette,[2] Bettina V. Lotsch,[2,3] and H. Martin R. Wilkening[1]

[1] Institute of Chemistry and Technology of Materials, Graz University of Technology (NAWI Graz), Stremayrgasse 9, Graz 8010, Austria

[2] Max Planck Institute for Solid State Research, Heisenbergstraße 1, 70569 Stuttgart, Germany

[3] LMU München, Butenandtstraße 5-13, 81377 Munich, Germany

E-mail: bernhard.gadermaier@tugraz.at

NMR analysis; assignment of the Na NMR lines. The β-like $Na_4P_2S_6$ phase has three different crystallographic Na sites, which are not fully resolved by MAS NMR likely due to the fast exchange between the Na2 and Na3 positions. These latter positions give one single coalesced signal in the ^{23}Na MAS NMR spectra at room temperature whereas the Na1 site produces a second line. From crystal-chemical considerations, the signal of the Na1 site cannot account for more than 50% of the total signal. Na1 fully occupies the 4g site, while Na2 and Na3 reside on 4h and 2d, respectively, with site occupation factors of approximately two-thirds (see Figure 1a). As the 4g site (Na1) is fully occupied, changes in the relative site occupation factors can only lead to a decrease in the Na1 signal, given the crystal-chemical limitations (Scholz et al.[1] wrt. structure). We performed different experiments at different Larmor frequencies and MAS rotor frequencies making use of the cooling and heating capabilities of the respective setups. To correctly assign the lines to the crystallographic positions, we cooled the sample down to –83 °C under MAS conditions to decrease the exchange rate between the Na2 and Na3 sites and to resolve the coalesced line (see Fig. S1a). These ^{23}Na MAS NMR spectra were recorded on a Bruker Avance-III wide bore spectrometer in a magnetic field of 9.4 T (Larmor frequency 105.8 MHz) using 4 mm ZrO_2 rotors in a BL4 MAS probe. Measurements were performed using the Bloch Decay excitation scheme with a total of 64 scans in each experiment. The temperature in the probe was regulated by using a Bruker BVT3000 temperature controller. The spectra were recorded using a very short excitation pulse of $\pi/24$ to ensure homogeneous excitation of the central transitions for sites with different quadrupolar coupling constants[2]. The spectra are referenced to the external signal of a 0.1 M solution of NaCl[3]. Neither line splitting nor a significant change in the chemical shift could be observed when the sample was cooled down to –83 °C. A much lower temperature could reveal which line is coalesced and hence stems from the Na2 and Na3 sites, but this is beyond the technical possibilities of our setup unless the sites are magnetically equivalent. Here, however, the relative intensities of the lines, obtained from fitting the spectra, indicate that the line located at lower ppm values stems from a smaller Na subpopulation, *i.e.* the Na1 position.

Supporting Information

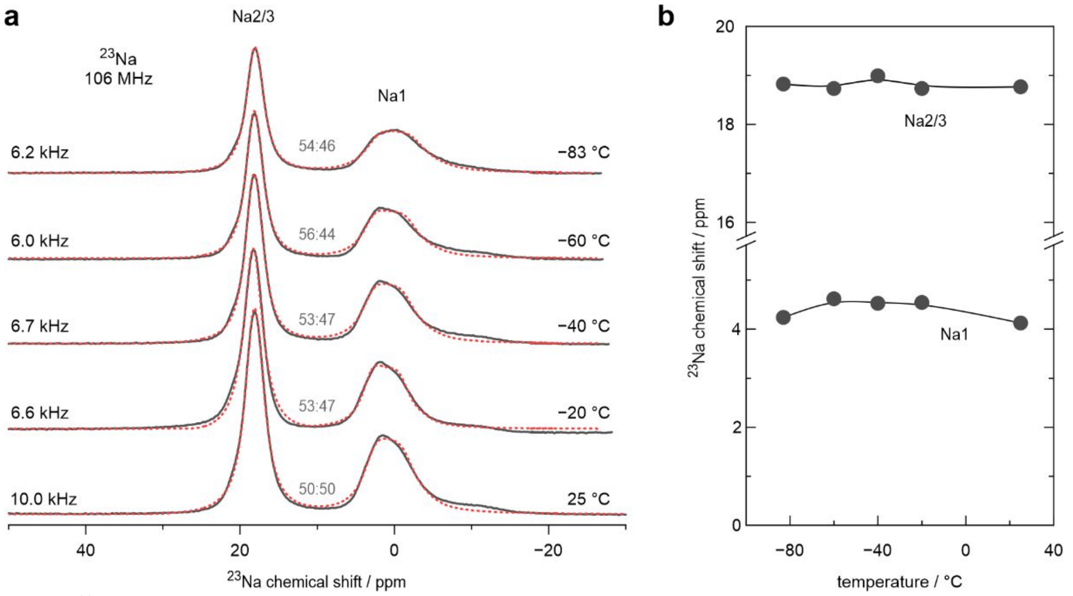

Figure S1: a) ^{23}Na MAS NMR spectra of $Na_4P_2S_6$ measured at various temperatures. The MAS rotor frequency, which varied due to cooling, is indicated. Ratios indicate the relative area fractions of the two lines from spectra fitting. The spectra are referenced to the ^{23}Na line of 1 M NaCl (0 ppm). b) ^{23}Na chemical shift as a function of temperature.

Using the laser-heating MAS NMR setup described in the methods section, the sample temperature was increased and spectra were recorded while the sample was spun at 4 kHz (see Fig. 1c and S2). From the relative area fractions of the observed lines, we attribute the signal at negative ppm values, referenced to solid NaCl, to the Na1 site since it accounts for only 42 % of the total integral signal (see Fig. S2). The coalesced line would be located exactly in the middle between the individual signals for a system with a 50:50 ratio of two different spin populations. Here, the coalesced line is, however, at a position slightly shifted towards the line located at positive ppm values again pointing to a slightly higher population of this line. Albeit there is a certain uncertainty, we assign the line appearing at negative ppm values to Na^+ on Na1 sites (4g). However, the following dynamic analysis would also hold if the lines were differently assigned.

NMR exchange rates from coalescence effects. To extract Na^+ exchange rates between the interlayer sites, we analyzed the ^{23}Na MAS NMR lines (i) by means of Lorentzian functions to extract line widths (full width half maximum, fwhm) and positions. We also (ii) took advantage of an analytical expression introduced by Reeves *et al.*,[4] which directly yields exchange rates. The spectra shown in Figure 1a were parametrized using Lorentzian functions. For the NMR responses recorded between 20 °C and 160 °C, the fits derived by using the analytical function are also shown. The Lorentzian functions indicate that both lines broaden as the temperature increases until they collapse into a single coalesced line (see Fig. S2b). This broadening is typical for lines if the exchange rate increases upon heating, as described by Gasparro *et al.*[5] The positions of the resonance signals experience a shift with increasing temperature (see inset in Figure S2b). At 180 °C, only a single line is observed, centered at a position slightly shifted towards the line associated with the higher spin population, *i.e.*, the Na2/3 line, see above.

The location of this coalesced line depends on the fractional populations of the spins located at ν_{Na1} and $\nu_{Na2/3}$. In general, the coalesced NMR signal is located at $\nu_{coalesc.} = P_{Na1}\nu_{Na1} + P_{Na2/3}\nu_{Na2/3}$ where P_{Na1} and $P_{Na2/3}$ represent the fractions of the populations which obey the relation $P_{Na1} + P_{Na2/3} = 1$. From the change of the positions, the exchange rate, k, can be determined according to the following relationship:

$$k = \pi \sqrt{\left(\Delta v_0^2 - \Delta v_T^2\right)/2},$$

where Δv_T^2 is the observed separation of the resonance lines on the frequency scale at any temperature. These exchange rates k are shown in Figure S2c (see triangles) and agree well with those derived from ^{23}Na SAE NMR, see circles. At the temperature of line coalescence, *i.e.*, at 180 °C, the exchange rate is in the order of the separation of the lines and can be determined via the expression $\tau_c^{-1} = \pi \Delta v_0$.[6] At only negligible exchange, v_0, the lines are separated by 3090 Hz in the present case. This value leads to an exchange rate $k \cong 10$ kHz at 180 °C (see square in Figure S2c). Using these three analysis approaches, *i.e.*, SAE NMR, (i) line convergence, and line coalescence analysis, the obtained exchange rates can be well analysed with an Arrhenius line, which yields an activation energy of 0.3 eV for the exchange process between the Na1 layer and the Na2/3 layer.

Complementarily, we used (ii) the analytical expression derived by Reeves *et al.*[4] The corresponding fits are shown in Figure S2a. This expression directly yields exchange rates, k, by taking the line convergence and the spin-spin relaxation time T_2 that governs line broadening, into account:

$$f(v) = Z + A\left\{\frac{\left((1/T_2)+2k\right)B + \left[v + (1-2P_A)\Delta v_0\right]C}{B^2 + C^2}\right\}$$

with $B = \frac{1}{T_2}\left((1/T_2)+2k\right) + \Delta v_0^2 - v^2$ and $C = 2\left[\left((1/T_2)+k\right)v - (1-2P_A)k\Delta v_0\right]$

Here, Z accounts for a linear background, A is a scaling factor, k is the exchange rate, and v is the frequency. This approach yields exchange rates (see crossed squares in Figure S2c) following Arrhenius behaviour with a rather high activation energy of 0.8 eV. NEB calculations by Rush and Holzwarth yield a similar migration energy of approx. 0.7 eV for the interlayer exchange process.[7]

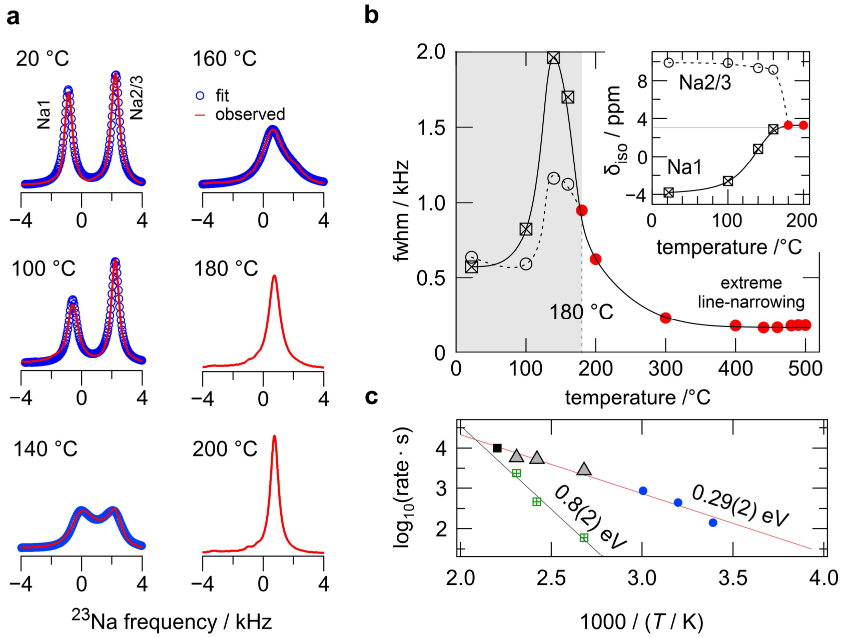

Figure S2 a) ^{23}Na MAS NMR lines recorded at indicated temperatures (line) and fit results using the analytical expression (circles) after Reeves *et al*. b) Evolution of the ^{23}Na MAS NMR linewidth (fwhm) as a function of temperature for the individual resonances up to 180 °C. The width of the coalesced line, seen above 180 °C, is also shown. The inset shows the change of the positions δ_{iso} of the NMR lines with temperature. The coalesced line slightly shifts towards the position of the line representing Na2/3. c) Exchange rates between the Na1 and Na2/3 layers determined from the line coalescence (filled square), line convergence (triangles), and SAE NMR (circles). Additionally, the rates extracted by fitting the analytical expression reported by Reeves *et al*. (see (a) for fits) are shown (crossed squares).

However, the fits using the analytical function indicate a strong temperature dependence of the relative spin populations which seems unreasonable while the higher activation energy seems plausible for a 1D diffusion process that connects the layers (see Figure 1a). Interestingly, both approaches are well in line with the exchange rate derived from the line-coalescence analysis (square in Figure S2c).

Approximating the Na jump frequency with the exchange rate between the layers, we can derive a corresponding diffusion coefficient for this 1D transport using the Einstein-relation $D_{NMR} \approx 0.5\, a^2\, k$. Here, the jump distances a lie between 0.36 nm (distance between the layers) and 0.48 nm (Na1─Na2 distance) and give rise to a diffusion coefficient at 180 °C in the range of 6 to 11×10^{-12} cm^2 s^{-1}. This characterizes a rather slow diffusion process.

NMR spin-lattice relaxation of ^{23}Na and ^{31}P. While the SLR rates of ^{23}Na are temperature-dependent over the whole temperature range from room temperature up to 650 °C the ^{31}P rates are only appreciable thermally activated above 300 °C. The ^{31}P rates are almost constant except for a sudden increase between 85 °C and 127 °C. However, at even higher temperatures, when the conversion to the gamma phase is completed by the Na-substructure and the emergence of the new ^{31}P signal becomes more pronounced (see Figure 2a), also the ^{31}P SLR is affected. The relaxation of the new signal accelerates quickly with temperature (blue circles in Figure 2d). We interpret this fast relaxation as further proof for the rotor phase suspected for γ-$Na_4P_2S_6$. See main text for further discussion.

For ^{23}Na, a different relaxation behavior is observed over this wide temperature window. The region of the β-phase is shown in Figure 1d. The relaxation rates, $1/T_1$, increase with temperature until a diffusion-induced rate peak at 202 °C is observed. Diffusion-induced rate peaks arise when the correlation rate τ_c^{-1} is in the order of the Larmor frequency, *i.e.,* $\omega_0 \cong \tau_c^{-1}$. Here, the jump rate, proportional to the inverse correlation time, reaches a value of 1.4×10^{-9} s^{-1}, which translates into a diffusion coefficient D_{NMR} of approximately 1.2×10^{-7} cm^2 s^{-1}. The asymmetric rate peak $1/T_1(1/T)$ yields an activation energy of 0.3(2) eV on the so-called low-T flank (see Figure 1d). This value was also found by Rush and Holzwarth for the in-plane, i.e. intralayer diffusion.[7]

Impedance spectroscopy up to 300 °C. The ionic conductivity was measured between −100 °C and 300 °C by using impedance spectroscopy. A single conduction process associated with a typical bulk capacitance of 50 pF was observed. The Arrhenius behavior describing the temperature dependence of the ionic conductivity is shown in Figure S3a. At temperatures below ca. 140 °C, the activation energy is 0.54 eV. Above 140 °C, the activation energy is slightly lower, 0.42 eV. Such effects might be caused by a change in the conduction mechanism and/or changes in the correlation of the jump processes. Variable-temperature XRPD measurements revealed a slight change of the thermal expansion coefficient at approx. the same temperature as the kink in the Arrhenius line, while the Raman measurements show no change. Possibly, the structural expansion eases the ionic transport process after a certain threshold, and hence the activation energy is decreased at higher temperature, see Figure S3a-b and below.

The measured specific conductivity σ_{DC} was converted into a Na-ion diffusion coefficient D by means of the Nernst-Einstein relation and the relation of the electric mobility with the specific conductivity: $D = (\sigma_{DC} T\, k_B)/(e^2 n)$ where T, k_B, e and n are the absolute temperature, the Boltzmann constant, the elementary charge, and the numeric charge carrier density, respectively. The latter was estimated based on the unit cell volume of the crystal structure and the number of Na ions in a unit cell.

Supporting Information

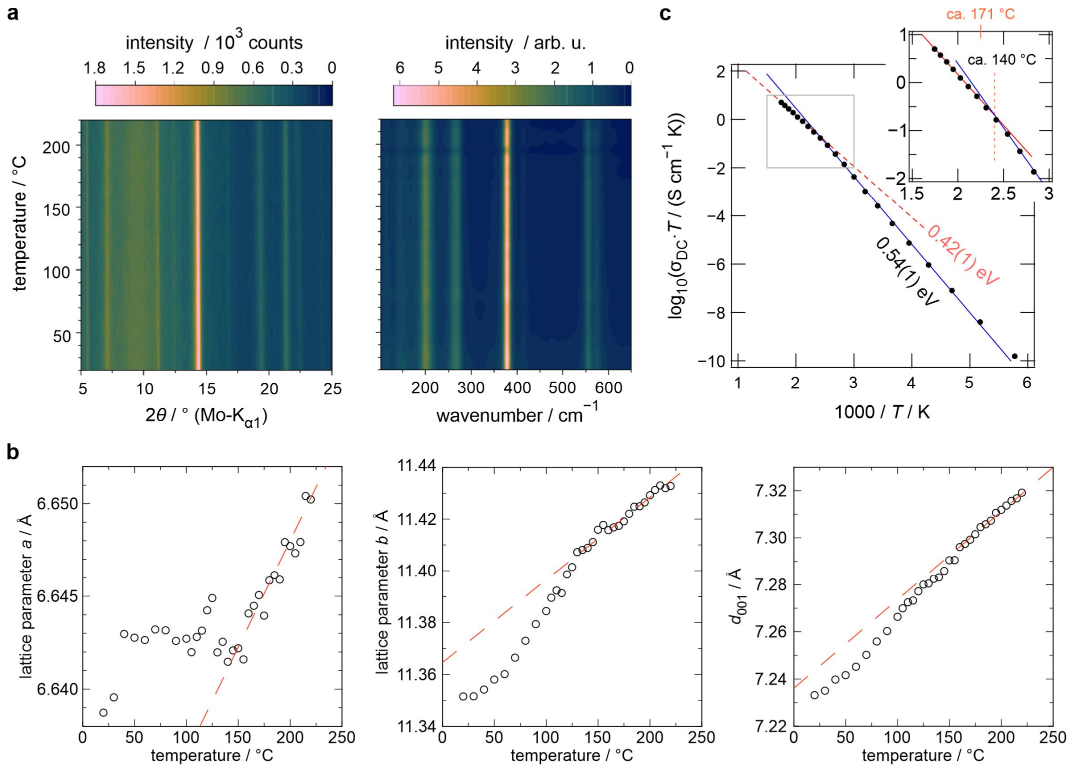

Figure S3 a) Contour plots of the X-ray diffraction data (left) and Raman spectra (right) of precipitated $Na_4P_2S_6$ recorded from ambient temperature up to 220 °C. b) Refined lattice parameters *a*, *b,* and the d_{001} spacing indicate a change of the thermal expansion coefficient at approx.140 °C. c) Arrhenius plot constructed from impedance data measured at temperatures between –100 °C and 300 °C for precipitated $Na_4P_2S_6$. A gradual change of slope is observed around 130 to 180 °C from 0.54(1) eV to 0.42(1) eV, see inset, which matches the observed change in the lattice parameters.

Side phase formation and reversibility. In Figure 2b, we show, that the transformation to the new Na environment of the γ-phase is completed at 590 °C. In Figure 3a, we show the ^{23}Na NMR spectra at temperatures up to 650 °C, where no further change in the line shape or position is observed. Starting from 610 °C, a minor signal is observed at low negative ppm values, see arrow in Figure 3a. The weak signal gets more intense with temperature and shows a small chemical shift. We assign this signal to a side phase formed at higher temperatures. No intensity is observed between the small signal and the line of $Na_4P_2S_6$, so we exclude exchange between the two Na environments.

To examine the reversibility of the phase formation, we measured the ^{23}Na NMR spectra also during the cool-down of the sample (see Figure 3b-c). We observed a hysteresis of the phase formation back to *β*-$Na_4P_2S_6$. However, all processes monitored during the heating of the sample are reversible when cooling down back to ambient. At 420 °C, the high-temperature side phase and the signal of the *γ*-$Na_4P_2S_6$ have disappeared. Also, the coalescence of the two signals of the low-temperature phase can be observed backward when cooling down the sample. These findings underline our interpretation of reversible phase transitions in $Na_4P_2S_6$ observed by MAS NMR.

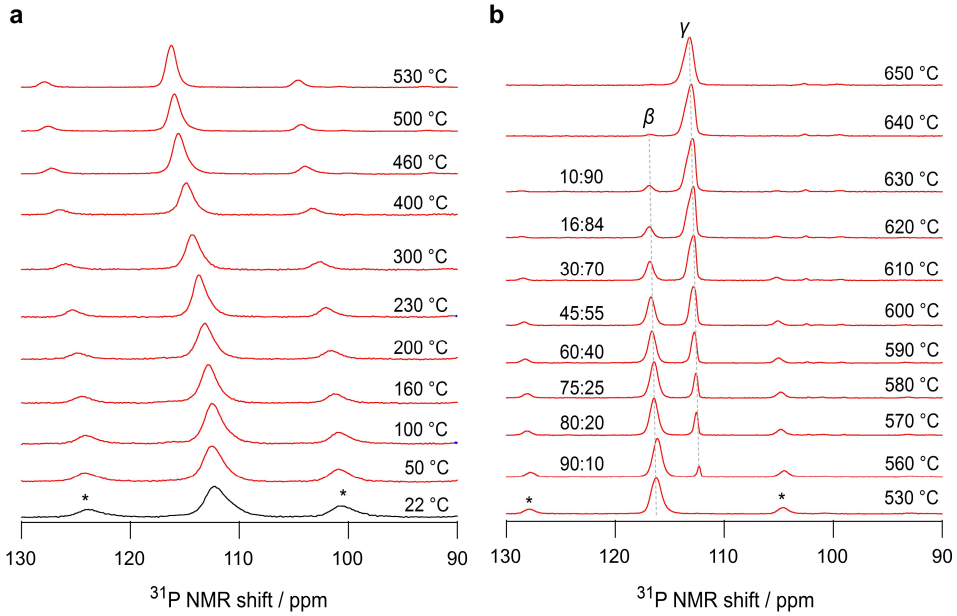

Figure S4 ^{31}P MAS NMR spectra showing the evolution of the phase-specific central transition lines during heating from 22 °C to 530 °C (a) and further to 650 °C (b). Asterisks denote spinning sidebands of the β-phase; the NMR line of the γ-phase shows almost no spinning side bands. a) Evolution of the ^{31}P NMR line during heating the β-phase from 22 °C to 530 °C. Except for a shift of the line, no significant changes are observed. b) During heating, the β-phase converts to the γ-phase while both phases coexist between 560 °C and 640 °C (heating run). Numbers indicate the relative intensities of the lines assigned to the β and the γ phase.

The same holds for the ^{31}P NMR spectra, cf. Figure S4 (see above). Here, we observe a minor signal at temperatures >600 °C at approx. 99 ppm. Accordingly, the side phase formed also includes phosphorus, but the formation is reversible as observed in ^{23}Na MAS NMR. In Figure S4a, we show the ^{31}P NMR spectra recorded at lower temperatures, from ambient up to 530 °C. In this temperature range, we see a small temperature-induced shift of the central transition, however, no change in the shape or width of the line spectra is detected. While the Na^+ environment changes drastically in this region, see main text, the local structure around P remains unchanged. Interestingly, the lines reveal no anomaly in the temperature range in which the ^{31}P MAS NMR SLR suddenly experienced a 4-fold increase (see Figure 2d).

Side bands were only observed for the peak associated with the β-phase but not for the γ-phase. This further underpins that the rotational motion of ^{31}P in the rotor phase is much higher. Phase fractions were determined by deconvoluting the spectra using two pseudo-Voigt functions for the central lines.

Although beyond the scope of this study, we recorded a few spectra at a constant temperature as a function of time to gain insight into the time-dependence of the phase transition. Figure S5a shows several ^{31}P MAS NMR spectra recorded at 560 °C after the sample's temperature was reduced from 580 °C.

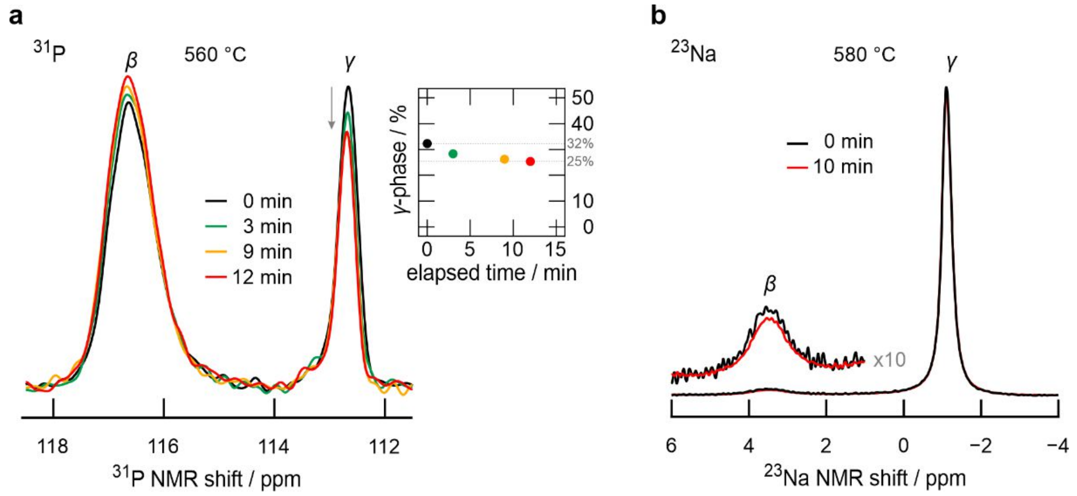

Figure S5 *a)* ^{31}P MAS NMR spectra recorded at 560 °C after cooling from 600 °C – the phase composition changes only slightly during the 12 min observation period. b) ^{23}Na spectra recorded at 580 °C after heating from 570 °C. When reaching the set temperature, and 10 minutes later, the β-phase amounts to 9% and 8%, respectively – a marginal difference in the scale of the uncertainty.

Even after keeping the sample at 560 °C for 12 min, the ^{31}P spectra reveal the presence of γ-$Na_4P_2S_6$. Similarly, the ^{23}Na spectra recorded at 580 °C also reveal the presence of both phases (see Fig. S5b)

We performed temperature-dependent XRPD measurements using a similar heating pattern as for the high-temperature MAS NMR measurements for complementary analyses. During heating between 400 °C and 650 °C (Fig. S6a) two major changes can be observed in the XRPD patterns. Until 600 °C, all Bragg reflections can be indexed by the monoclinic unit cell of β-$Na_4P_2S_6$. Below 480 °C some of these reflections, like 110, $11\bar{1}$ or $22\bar{1}$ exhibit strong anisotropic broadening, indicating the presence of planar defects, *i.e.* stacking fault disorder. At temperatures above 480 °C, the reflections gradually become sharper (Fig. S6b, red indices), indicating a healing of the stacking faults. This irreversible healing of defects corroborates the observation of a narrowed ^{23}Na MAS NMR line observed after the sample was subjected to temperatures up to 650 °C (see Fig. 3c). Reflections, assigned to cubic γ-$Na_4P_2S_6$ (Fig. S6c, red indices) appear at 600 °C and gradually become more intense upon further heating.

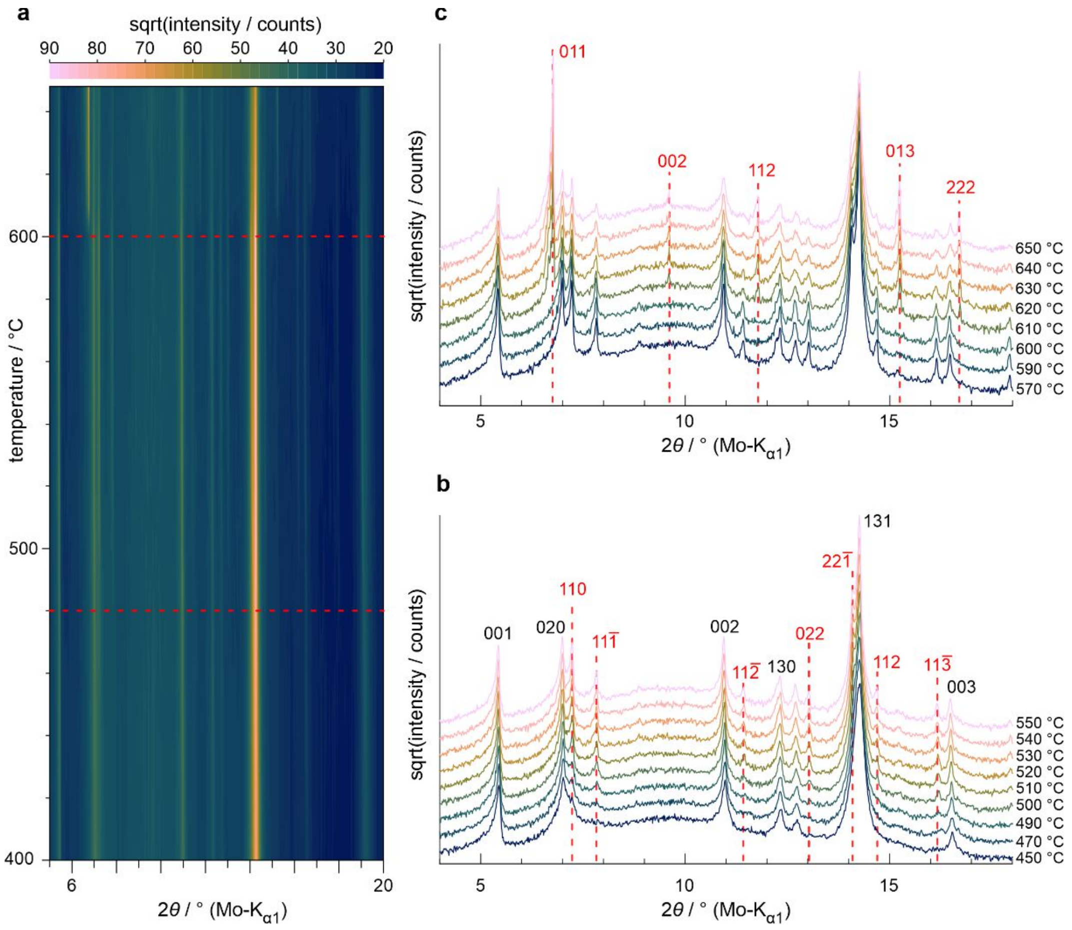

Figure S6 a) Heatmap of *in-situ* powder X-ray diffraction pattern of $Na_4P_2S_6$, measured from 400 °C to 650 °C. Initial temperature increments were set at 400 °C, 450 °C, 470 °C, and 490 °C. Beyond 500 °C, the increments were adjusted to steps of 10 K. Two major changes in the diffractograms can be observed at 480 °C and 610 °C (see dashed lines). In b) and c), these changes are resolved as stacked diffractograms. b) Stacked XRPD pattern of β-$Na_4P_2S_6$ measured between 450 °C and 550 °C. The reflections already observed at low temperatures are indexed in black, while the additional reflections evolving from diffuse scattering with increasing temperature are indexed in red and marked with a dotted red line. c) Stacked XRPD pattern of $Na_4P_2S_6$ measured between 570 °C and 650 °C. The increasing reflections of the cubic γ-phase are indexed in red and marked with a dotted red line.

^{31}P MAS NMR indicates that a 50:50 ratio of the beta and gamma phase is present at this temperature (see Fig. 2a).

Supporting Information

References

1. Scholz, T.; Schneider, C.; Eger, R.; Duppel, V.; Moudrakovski, I.; Schulz, A.; Nuss, J.; Lotsch, B. V., Phase formation through synthetic control: polymorphism in the sodium-ion solid electrolyte $Na_4P_2S_6$. *Journal of Materials Chemistry A* **2021,** *9* (13), 8692-8703.
2. Freude, D.; Haase, J., Quadrupole Effects in Solid-State Nuclear Magnetic Resonance. In *Special Applications*, Pfeifer, H.; Barker, P., Eds. Springer Berlin Heidelberg: Berlin, Heidelberg, 1993; pp 1-90.
3. Harris, R. K.; Becker, E. D.; Menezes, S. M. C. d.; Goodfellow, R.; Granger, P., NMR nomenclature. Nuclear spin properties and conventions for chemical shifts (IUPAC Recommendations 2001). *Pure and Applied Chemistry* **2001,** *73* (11), 1795-1818.
4. Reeves, L. W.; Shaw, K. N., Nuclear magnetic resonance studies of multi-site chemical exchange. I. Matrix formulation of the Bloch equations. *Canadian Journal of Chemistry* **1970,** *48* (23), 3641-3653.
5. Gasparro, F. P.; Kolodny, N. H., NMR determination of the rotational barrier in N,N-dimethylacetamide. A physical chemistry experiment. *Journal of Chemical Education* **1977,** *54* (4), 258.
6. Bowman, R. C.; Adolphi, N. L.; Hwang, S.-J.; Kulleck, J. G.; Udovic, T. J.; Huang, Q.; Wu, H., Deuterium site occupancy and phase boundaries in $ZrNiD_x$ ($0.87 \leq x \leq 3.0$). *Physical Review B* **2006,** *74* (18), 184109.
7. Rush, L. E.; Holzwarth, N. A. W., First principles investigation of the structural and electrochemical properties of $Na_4P_2S_6$ and $Li_4P_2S_6$. *Solid State Ionics* **2016,** *286*, 45-50.
